# Temporal and Spatial Analysis of Alzheimer’s Disease Based on an Improved Convolutional Neural Network and a Resting-State FMRI Brain Functional Network

**DOI:** 10.3390/ijerph19084508

**Published:** 2022-04-08

**Authors:** Haijing Sun, Anna Wang, Shanshan He

**Affiliations:** 1 College of Information Science and Engineering, Northeastern University, Shenyang 110819, China; seamirror@126.com (H.S.); shanshanhe.neu@foxmail.com (S.H.); 2 College of Intelligent Science and Engineering, Shenyang University, Shenyang 110044, China

**Keywords:** convolutional neural network, resting-state fMRI, brain functional network, AD diagnostic, MCI transformation prediction

## Abstract

Most current research on Alzheimer’s disease (AD) is based on transverse measurements. Given the nature of neurodegeneration in AD progression, observing longitudinal changes in the structural features of brain networks over time may improve the accuracy of the predicted transformation and provide a good measure of the progression of AD. Currently, there is no cure for patients with existing AD dementia, but patients with mild cognitive impairment (MCI) in the prodromal stage of AD dementia may be diagnosed. The study of the early diagnosis of MCI and the prediction of MCI to AD transformation is of great significance for the monitoring of the MCI to AD transformation process. Despite the high rate of MCI conversion to AD, the neuropathological cause of MCI is heterogeneous. However, many people with MCI remain stable. Treatment options are different for patients with stable MCI and those with underlying dementia. Therefore, it is of great significance for clinical practice to predict whether patients with MCI will develop AD dementia. This paper proposes an improved algorithm that is based on a convolution neural network (CNN) with residuals combined with multi-layer long short-term memory (LSTM) to diagnose AD and predict MCI. Firstly, multi-time resting-state fMRI images were obtained from the Alzheimer’s Disease Neuroimaging Initiative (ADNI) database for preprocessing, and then an AAL brain partition template was used to construct a 90 × 90 functional connectivity (FC) network matrix of a whole-brain region of interest (ROI). Secondly, the diversity of training samples was increased by generating an adversarial network (GAN). Finally, a CNN with residuals and a multi-layer LSTM model were constructed to automatically classify and predict the functional adjacency matrix. This method can not only distinguish Alzheimer’s disease from normal health conditions at multiple time points, but can also predict progressive MCI (pMCI) and stable MCI (sMCI) at multiple time points. The classification accuracies in AD vs. NC and sMCI vs.pMCI reached 93.5% and 75.5%, respectively.

## 1. Introduction

Alzheimer’s disease (AD) is a common, non-reversible, and progressive neurological disease characterized by cognitive impairment, with patients’ memory and thinking abilities gradually becoming impaired over time [1]. Mild cognitive impairment (MCI) is a transitional stage between normal aging and AD and is characterized by mild memory and intellectual impairment, with a degree of memory impairment not commensurate with age [2]. MCI is a preclinical risk factor for AD. The conversion of MCI to AD is ten times more common than in the general population. According to follow-up studies, the incidence of transition to AD in patients with MCI is 10 to 15% within 1 year, 40% within 2 years, and 20 to 53% within 3 years [3]. Therefore, MCI patients can be further divided into stable MCI (sMCI) and progressive MCI (pMCI) patients. Although there is no treatment for MCI and AD patients at present, the study on the early diagnosis of MCI and the prediction of MCI to AD transformation is of great significance for the monitoring of the MCI to AD transformation process [4].Research on the diagnosis of AD and the prediction of the MCI–AD transformation process has become a hotspot for current scholars. With the development of medical neuroimaging technology, magnetic resonance imaging (MRI) has provided more effective data support for related scholars to study AD due to its safety, non-invasiveness, high pixel resolution, and flexible imaging methods [5]. In the research on AD and the prediction of the MCI–AD transformation process based on resting-state functional MRI (rs-fMRI) data, the most common method used is to abstract the pictorial data into numerical data and construct functional brain adjacency networks. The study of the differences in functional brain adjacency networks between subjects with AD and normal subjects, as well as between subjects with sMCI and pMCI, has become a hot topic in recent years. As a potential biomarker, the functional connectivity (FC) matrix has attracted much attention in many studies [6]. In recent years, strategies for dementia diagnosis based on deep learning methods have achieved good results over traditional machine learning methods because deep learning models can extract the differential feature representation hierarchically and can naturally combine features of different levels together [7]. Most domestic and foreign research is based on deep learning for horizontal classification diagnosis, such as normal control groups (NC) and AD dichotomies or NC, MCI, and AD tri-classification studies [8]. Zhang et al. [9] studied the functional connectivity of the whole brain by calculating Pearson’s correlation coefficients based on rs-fMRI data and proposed a set of novel features by applying the two-sample *t*-test on the correlation coefficients’ matrix to identify the most discriminative correlation coefficients. Taie et al. [10] proposed a bat-based support vector machine (SVM) parameter optimization model for the diagnosis of AD in MRI bio-medical images. The model uses MRI to classify biomedical images and diagnose three kinds of biomedical images: NC, MCI, and AD. Xu et al. [11] proposed a new deep learning method called the multiple graph Gaussian embedding model (MG2G), which maps high-dimensional resting-state brain networks to low-dimensional latent spaces to learn information-rich network features. This model predicts the progression to AD in patients with MCI and identifies altered areas of the brain network associated with MCI. According to research, the essence of AD deterioration is the degeneration of brain nerve function. Therefore, by observing and recording the longitudinal changes of brain functional links over time, one can detect AD in advance and take timely intervention measures to prevent the occurrence of AD or slow down the rate of neurodegeneration. In the longitudinal study, data collected at multiple time points will be involved [12]. Longitudinal data capture the progression of disease dynamics as opposed to data at a single point in time. In this paper, convolutional neural networks (CNN) and recursive neural networks (RNN) with long short-term memory (LSTM) are introduced into the whole-brain functional networks analysis. A longitudinal joint analysis method of brain functional networks based on deep learning is proposed. In this method, rs-fMRI images of NC, sMCI, pMCI, and AD at baseline, 12 months, and 24 months were screened from the ADNI database to construct and analyze the brain functional networks [13]. During the progression from MCI to AD, the connectivity patterns between brain regions change, and the information transmission ability and efficiency of the brain’s functional networks are impaired. In this paper, the whole-brain fMRI functional network connection was selected as a marker to carry out the study [14]. The connection characteristics of the whole-brain functional networks were extracted and combined with the longitudinal characteristics to classify NC and AD and predict whether MCI patients were developing AD [15]. The proposed method was validated in ADNI data sets and compared with other classical methods. The results show that the proposed method has a higher classification and prediction accuracy and stronger robustness than traditional methods.

## 2. Materials and Methods

### 2.1. Data Selection

The sample data used in this study were from the Alzheimer’s Disease Neuroimaging Initiative (ADNI) (http://adni.loni.usc.edu, accessed on 12 January 2022). There were 312 subjects in this study, including 100 for NC, 75 for sMCI, 72 for pMCI, and 65 for AD. fMRI images were collected at baseline (BL), 12 months, and 24 months in each group. Prior to the scan, the subjects passed cognitive and behavioral assessments [16]. Sample demographic information is shown in Table 1.

**Figure 1:**
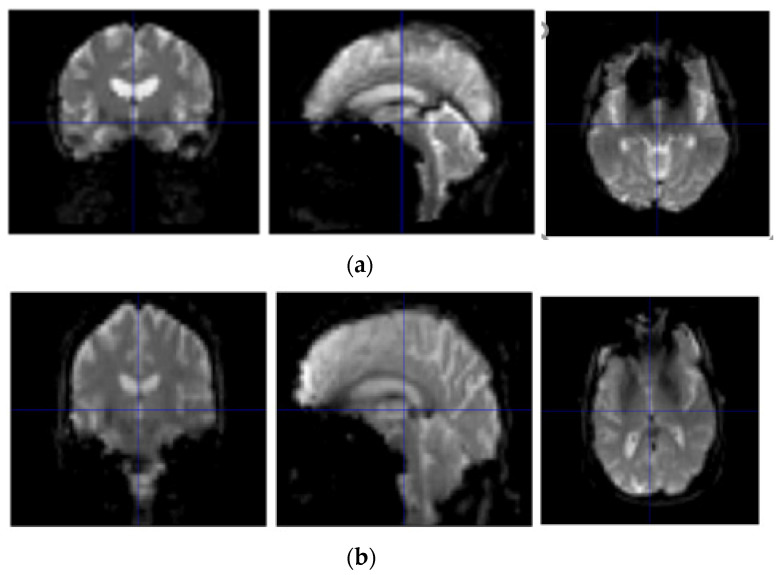

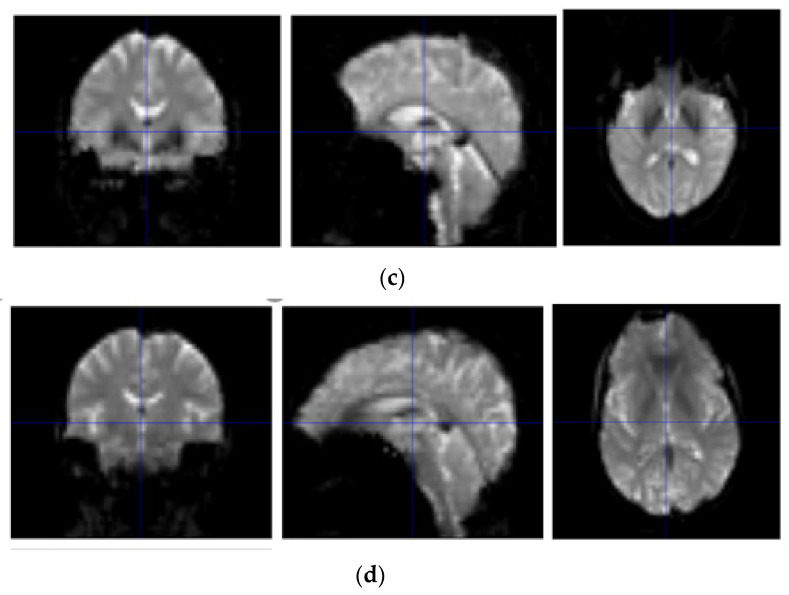
Resting-state fMRI image display of NC, AD, sMCI, and pMCI subjects. (**a**) Resting-state fMRI image display of a NC subject; (**b**) resting-state fMRI image display of an AD subject; (**c**) resting-state fMRI image display of a sMCI subject; and (**d**) resting-state fMRI image display of a pMCI subject.

As can be seen from Table 1, with the aggravation of the disease, MMSE scores showed a downward trend, while CDR showed an upward trend. A statistical analysis of basic information was obtained by SPSS software [17]. Sample scanners selected from the ADNI are from Philips Medical Systems. The resting state fMRI scan sequence (EPI) has a total of 140 time points with 48 layers, a magnetic field intensity of 3.0 tesla, a flip angle of 80.0, a TE of 30.0 ms, a TR of 3000.0 ms, a 64 × 65 matrix, and 6720.0 images with a thickness of 3.31 mm. The resting-state fMRI image display of NC, AD, sMCI, and pMCI subjects is shown in Figure 1.

### 2.2. Image Preprocessing

**Figure 2:**
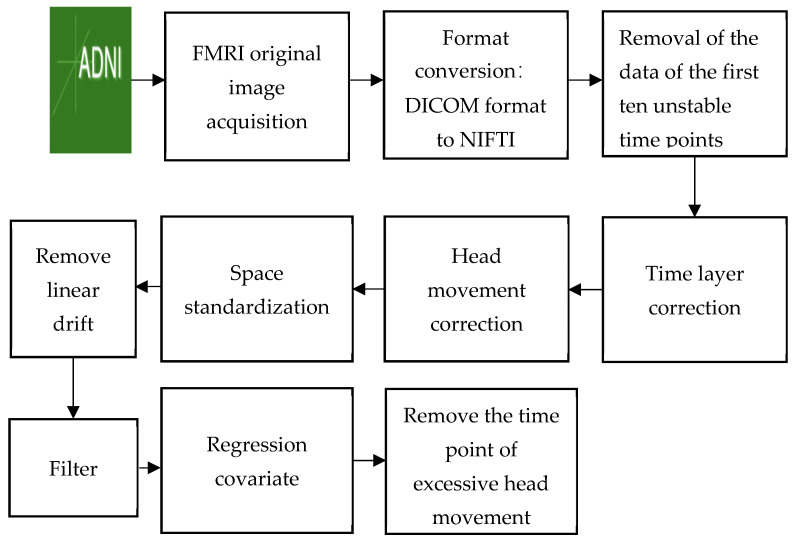
Pretreatment flow chart.

The process of fMRI data preprocessing includes data format conversion, the removal of unstable time points, time layer correction, head movement correction, spatial standardization, the removal of linear drift, filtering, regression covariates, and the removal of excessive head movement time points [18]. In this paper, the preprocessing process is basically the same as that of general MRI, but the difference is that the preprocessing in this paper does not need to be performed smoothly because network analysis requires high spatial accuracy, and smoothness will affect the activation of adjacent regions of interest (ROIs). The pretreatment process of fMRI data includes format conversion (DICOM format to NIFTI), the removal of the first ten unstable time points, time layer correction, head correction, spatial standardization, linear drift removal, filtering, regression covariates, and the removal of excessive head movement time points (in order to reduce the influence of head movements and artifacts, subjects with FD > 0.5 over 2.5 min (50 frames) of data were excluded). The SPM8 toolbox and the DPARSFA (version 2.2) toolkit were used for standard preprocessing [19,20]. The pretreatment flow chart is shown in Figure 2.

### 2.3. Whole-Brain Functional Link Matrix FC

The functional connectivity of the human brain is complex, and the connectivity of functional brain networks has been widely used in the study of AD. The construction of brain functional networks based on fMRI data was mainly divided into the following steps [21,22]:

(1) Nodes (brain regions) were obtained, and the whole brain was divided into 90 ROI brain regions using the AAL (AAL90) template. Once the partitioning method was selected, the node was identified.

(2) The whole-brain functional connectivity matrix was obtained using fMRI data and nodes. We averaged the voxels in each ROI brain region, obtained fMRI time series signals in each ROI brain region, and constructed the brain functional network of each subject by calculating the Pearson correlation coefficient between two ROIs. The Pearson correlation coefficients of the two time series are shown in Equation (1):

(1) $$\rho_{X,Y}=\frac{Cov\left(X,Y\right)}{\sigma_{X}\sigma_{Y}}=\frac{E\left(\left(X-\mu_{X}\right)\left(Y-\mu_{Y}\right)\right)}{\sigma_{X}\sigma_{Y}}$$

**Figure 3:**
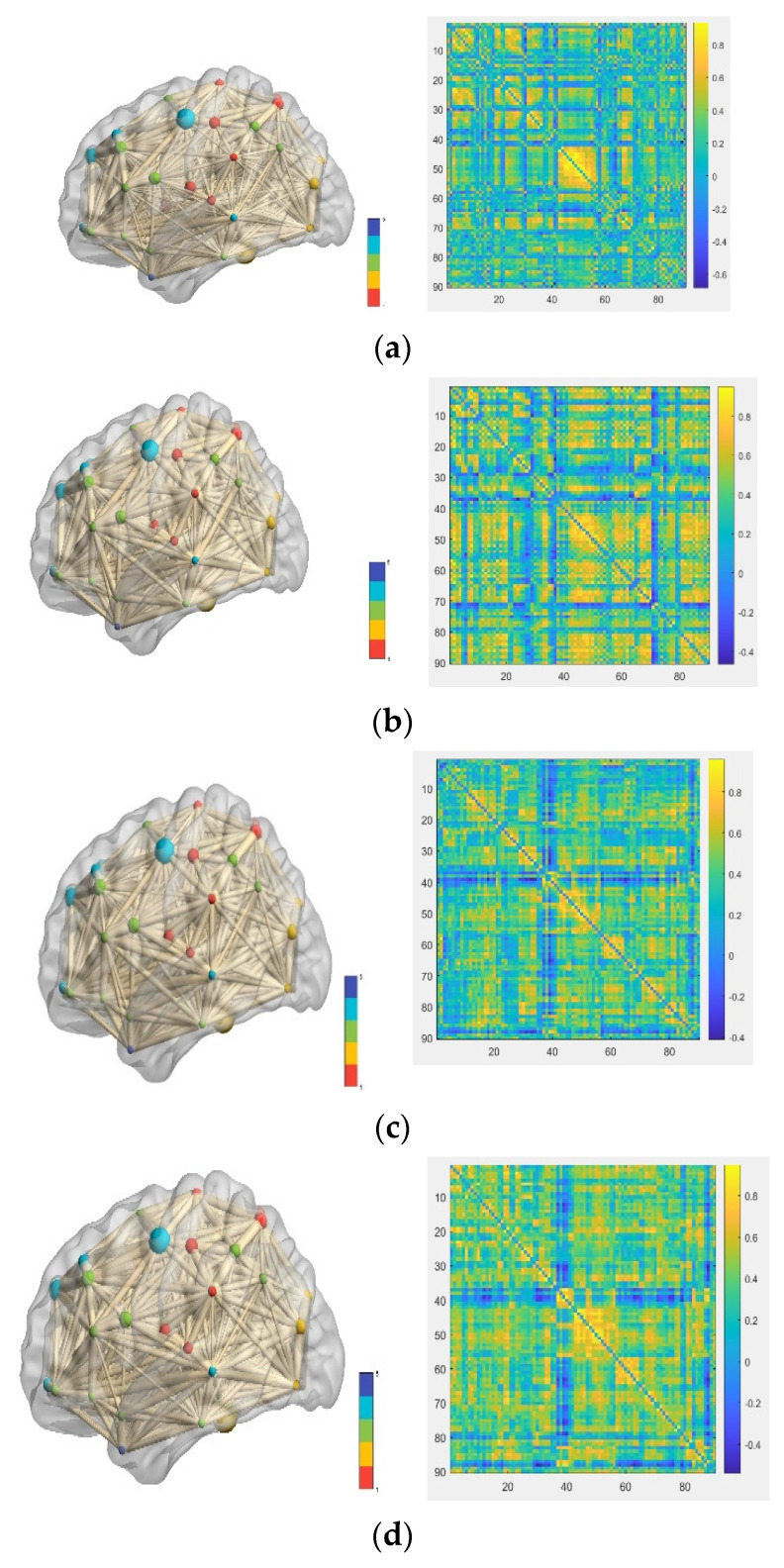
Brain network visualization and functional connection matrix. (**a**) Brain network visualization and the functional connection matrix of N; (**b**) brain network visualization and the functional connection matrix of AD; (**c**) Brain network visualization and the functional connection matrix of sMCI; and (**d**) Brain network visualization and the functional connection matrix of pMCI.

This is the product of the covariance of the two time series divided by the standard deviation of the two time series. The results of the Pearson correlation coefficient $\rho_{X,Y}$ are in the range of $-1\leq \rho_{X,Y}\leq 1$. When $0\leq \rho_{X,Y}\leq 1$, the two time series are positively correlated or they are negatively correlated. When it equals zero, it means that the two time series are independent of each other and have no correlation. From this, it can be concluded that the functions of two groups of brain areas in a certain period of time are synergistic or antagonistic. By calculating the average time series of each brain region and calculating the correlation coefficient in pairs, the correlation matrix of the whole brain during this period of time can be obtained, namely the functional connection matrix [23,24,25]. The functional connectivity matrix is displayed using the AAL90 template with a total of 90 brain regions, so the connectivity matrix is 90 × 90. Brain network visualization and the functional connection matrix are shown in Figure 3.

### 2.4. The Improved Method Proposed in This Paper

At present, common brain image analysis methods to manually extract the specified features are mainly based on prior knowledge, which results in great limitations in the representation of image features. Most brain image analysis studies are focused on image data analysis at a single time point, which is prone to interference from different individuals. Longitudinal image data analysis at multiple time points in the time domain can obtain pathological changes in the pathogenesis process and achieve a more precise diagnosis of Alzheimer’s disease [26]. Aiming at the above problems, this paper proposes an automatic analysis and diagnosis model of the multi-temporal brain function network based on the deep learning method. The functional connectivity (FC) (90 × 90) between brain ROI regions was used as the original input feature of CNNs. As deep neural networks generally require a large amount of training data to obtain ideal results, in the case of limited data in this paper, it is necessary to build a GAN to perform data augmentation for samples [27]. Then, a 1D−CNN model was built to extract spatial features. Then, a three-layer LSTM model was built to extract and analyze FC features at multiple time points [28,29]. Finally, the validity of the model was verified against the ADNI data set.

(1) GAN based data augmentation

**Figure 4:**
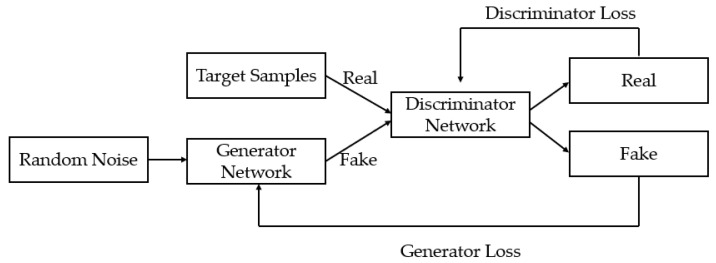
The principal diagram of data augmentation by GAN.

The GAN model contains two networks: one is a generative network, and the other is an adversarial network. The role of the generative network is to generate new samples in the case of given samples, so that the adversarial network cannot distinguish between these new samples and given samples. Therefore, GAN is generally a model that can generate synthetic samples that can reflect the target distribution behind real data and achieve the purpose of data augmentation [30]. The principal diagram of data augmentation by GAN is shown in Figure 4.

(2) Spatial feature extraction based on CNNs

**Figure 5:**
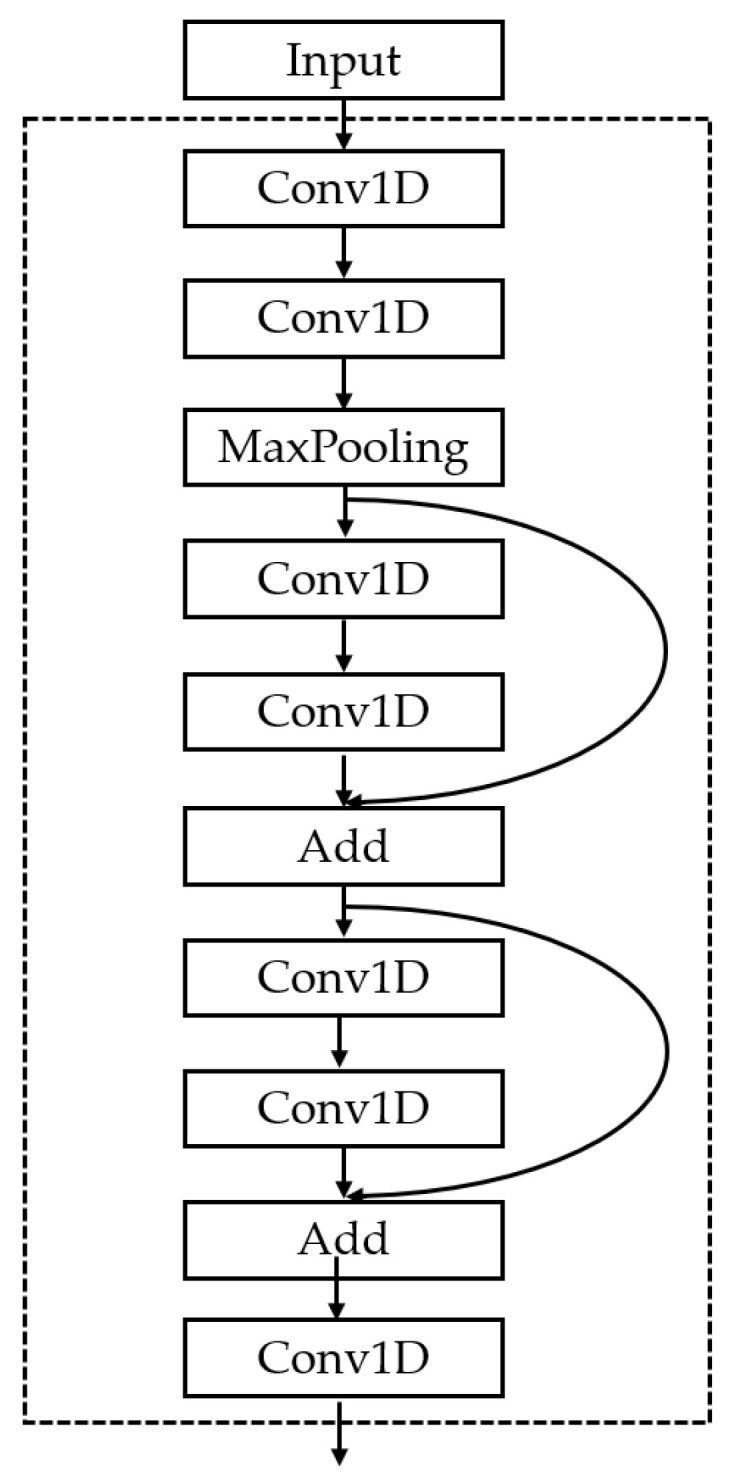
The 1D-CNN model structure.

Convolutional neural networks (CNN) are very similar to common neural networks in that they are both made up of neurons with learnable weights and biases. Every neuron takes some input and generates some dot products, and the output is the fraction of each classification [31]. The function of the convolution layer is feature extraction. For the brain’s functional network at each time point, we built a 1D-CNN model with the same structure to extract spatial features at a single time point [32]. The model structure is shown in Figure 5.

The model includes operations, such as convolution, max-pooling, and short connection structure. In this paper, the traditional CNN model with a single direction and vertical structure is improved. In the improved model, two short connection modules are added to fuse the features of the front and rear layers and enhance the utilization of the front layer [33].

(3) RNN

**Figure 6:**
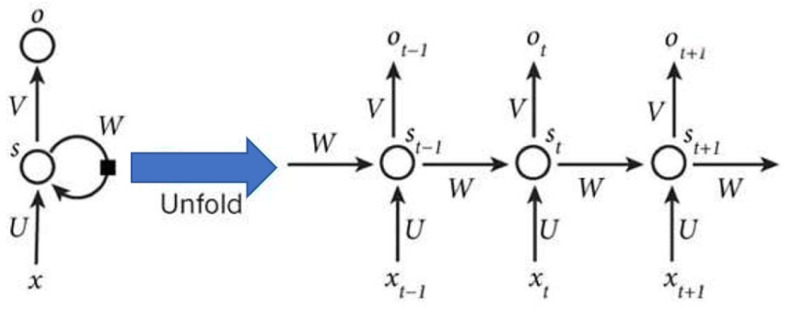
Diagram of the expansion of a recurrent neural network.

Recurrent neural networks (RNNs) have achieved great success and are widely used in many natural language processing (NLP) applications. RNNs are mainly used to process sequence data. A simple RNN consists of an input layer, a hidden layer, and an output layer [34]. The RNN can be expanded using a timeline, as shown in Figure 6.

In Figure 6, there is a one-way flow of information from the input unit to the hidden unit, and another one-way flow of information from the hidden unit to the output unit. In some cases, the RNNs break the latter restriction, guiding information from the output unit back to the hiding element. These are called “back projections,” and the input to the hiding layer also includes the status of the upper hiding layer, where nodes can be self-connected or interconnected [35].

In Figure 6, after the network receives the input $x_{t}$ at time *t*, the value of the hidden layer is $s_{t}$ and the output value is $o_{t}$. The key point is that the value of $s_{t}$ not only depends on $x_{t}$, it depends on $s_{t-1}$. The calculation method of recurrent neural networks can be expressed as shown in Equations (2) and (3):

(2) $$o_{t}=g(Vs_{t})$$

(3) $$s_{t}=f(Ux_{t}+Ws_{t-1})$$

It can be seen from Equations (2) and (3) that the difference between the cyclic layer and the fully connected layer is that the cyclic layer has a weight matrix W. If Equation (3) is repeatedly substituted into Equation (2), Equation (4) will be obtained:

(4) $$\begin{array}{ll} o_{t} & =g(Vs_{t})\\ & =g(Vf(Ux_{t}+Ws_{t-1}))\\ & =g(Vf(Ux_{t}+W(f(Ux_{t-1}+Ws_{t-2})))\\ & =g(Vf(Ux_{t}+W(f(Ux_{t-1}+W(f(Ux_{t-2}+Ws_{t-3}))))\\ & =g(Vf(Ux_{t}+W(f(Ux_{t-1}+W(f(Ux_{t-2}+W(f(Ux_{t-3}+\ldots ))))) \end{array}$$

RNNs have problems with gradient disappearance and gradient explosion in the process of long sequence training, i.e., information loss caused by long-distance transmission.

(4) LSTM

**Figure 7:**
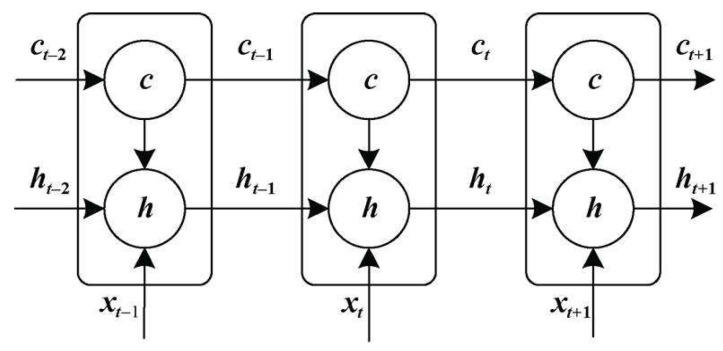
LSTM is expanded in time dimensions.

The long memory network (LSTM) successfully solved the defects of the original recurrent neural network and became the most popular RNN at present. It has been successfully applied in many fields, such as speech recognition, image description, and natural language processing. The hidden layer of the original RNN has only one state, H, which is very sensitive to short-term input. Thus, let us add another state C, to preserve the long-term state [36]. This is shown in Figure 7:

The forgetting gate is shown in Equation (5):

(5) $$f_{k}=\sigma \left(W_{f}\cdot \left[h_{k-1},x_{k}\right]+b_{k}\right),k=t,t+1,t+2$$

$W_{f}$ an be written as Equation (6):

(6) $$\begin{array}{ll} \left[W_{f}\right]\left[\begin{array}{c} h_{k-1}\\ x_{k} \end{array}\right] & =\left[W_{fh}W_{fx}\right]\left[\begin{array}{c} h_{k-1}\\ x_{k} \end{array}\right]\\ & =W_{fh}h_{k-1}+W_{fx}x_{k},k=t,t+1,t+2 \end{array}$$

The input gate is shown in Equation (7):

(7) $$i_{k}=\sigma \left(W_{i}\cdot \left[h_{k-1},x_{k}\right]+b_{i}\right),k=t,t+1,t+2$$

In the above formula, $W_{i}$ is the weight matrix of the input gate, and $b_{i}$ is the bias term of the input gate [37,38].

Next, the cell state ${\tilde{c}}_{k}$ used to describe the current input is calculated based on the previous output, and the current input is shown in Equation (8):

(8) $${\tilde{c}}_{k}=\tanh\left(W_{c}\cdot \left[h_{k-1},x_{k}\right]+b_{c}\right),k=t,t+1,t+2$$

This equation calculates the cell state $c_{t}$ at the current time. It is produced by multiplying the element of the last cell state $c_{t-1}$ by the forgetting gate $f_{t}$, and then multiplying the element of the current input cell state ${\tilde{c}}_{t}$ by the input gate ${\tilde{c}}_{t}$, and then adding the two products shown in Equations (9) and (10):

(9) $$c_{k}=f_{k}c_{k-1}+i_{k}{\tilde{c}}_{k},k=t,t+1,t+2$$

(10) $$o_{k}=\sigma \left(W_{o}\left[h_{k-1},x_{k}\right]+b_{o}\right),k=t,t+1,t+2$$

The final output of LSTM is determined by the output gate and cell state shown in Equation (11):

(11) $$h_{k}{=o}_{k}\tanh\left(c_{k}\right),k=t,t+1,t+2$$

**Figure 8:**
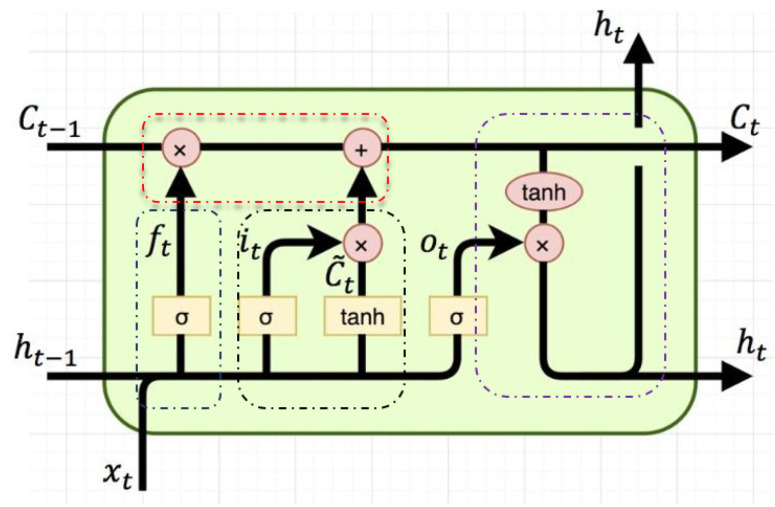
Final calculation diagram of LSTM.

**Figure 9:**
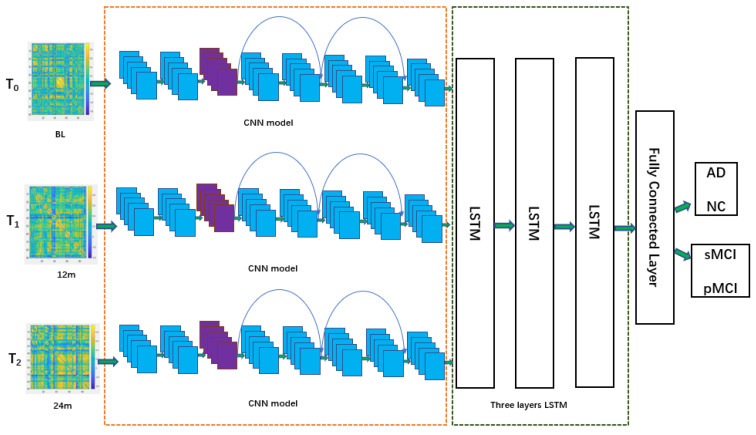
The design of a CNN combined with a three-layer LSTM framework.

Figure 8 shows the calculation of the final output of LSTM:

In this paper, we set up a three-layer LSTM model to analyze these sequences and extract the temporal variation characteristics of the spatial features at different time points, so as to make comprehensive use of single-time point and multi-time point information to diagnose and predict AD [39,40]. The design of a CNN combined with the three-layer LSTM framework is shown in Figure 9.

**Figure 10:**
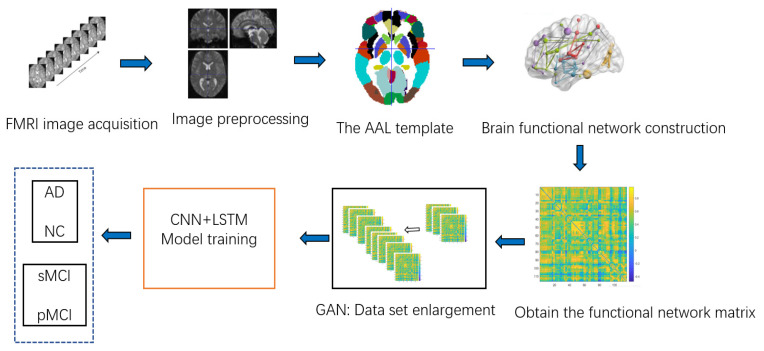
The overall framework for AD diagnosis and MCI prediction.

The overall framework for AD diagnosis and MCI prediction is shown in Figure 10.

**Figure 11:**
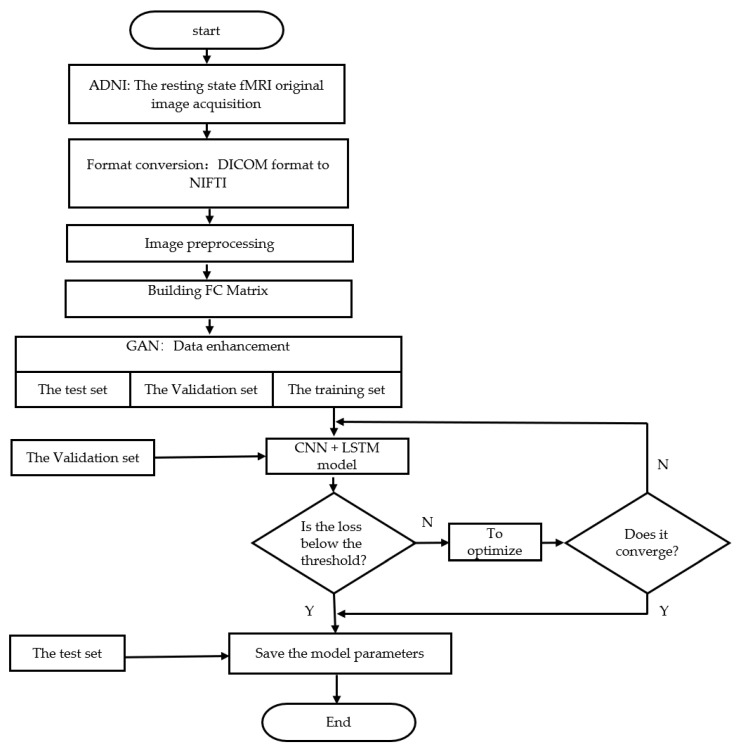
The overall program flow chart of this model.

The overall program flow chart of this model is shown in Figure 11.

## 3. Experimental Result

After the format conversion and image preprocessing of the original fMRI data obtained from ADNI in the experiment, the CNN model and LSTM model in this algorithm were built in the Python environment with the help of the deep learning library Keras and TensorFlow. The hardware configuration of this experiment is as follows: 8-core, 16-thread, AMD R7-4800U CPU, 16 G memory, 512 G hard disk, and a 4.2 GHz acceleration frequency. We performed an experimental test of the proposed multi-time resting-state fMRI brain functional network study on the ADNI database. We divided the whole data set into five parts, selected four pieces at a time as the training set, with the remaining one as the test set, and randomly selected part of the training set as the verification set. We used the accuracy, precision, and recall rate to evaluate the effect of this classification. The accuracy, precision, and recall rate are shown as Equation (12), Equation (13), and Equation (14), respectively.

(12) $$Accuracy=\frac{TP+TN}{TP+TN+FN+FP}100\%$$

(13) $$Precision=\frac{TP}{TP+FP}100\%$$

(14) Recall=TPTP+FN100%

where *TP* means the prediction is positive, and the reality is positive;

*TN* means the prediction is negative, and the reality is negative;

*FP* means the prediction is positive, and the reality is negative; and

*FN* means the prediction is negative, and the reality is positive.

**Figure 12:**
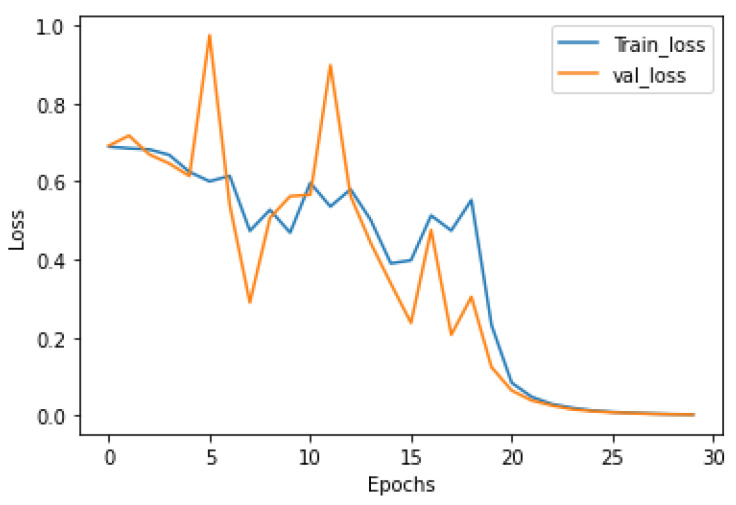
The loss curve.

The loss curve is shown in Figure 12, and the experimental results based on the convolutional neural network and resting state fMRI brain functional network are shown in Table 2. The blue line represents the loss curve of the training set, and the orange line represents the loss curve of the validation set.

**Figure 13:**
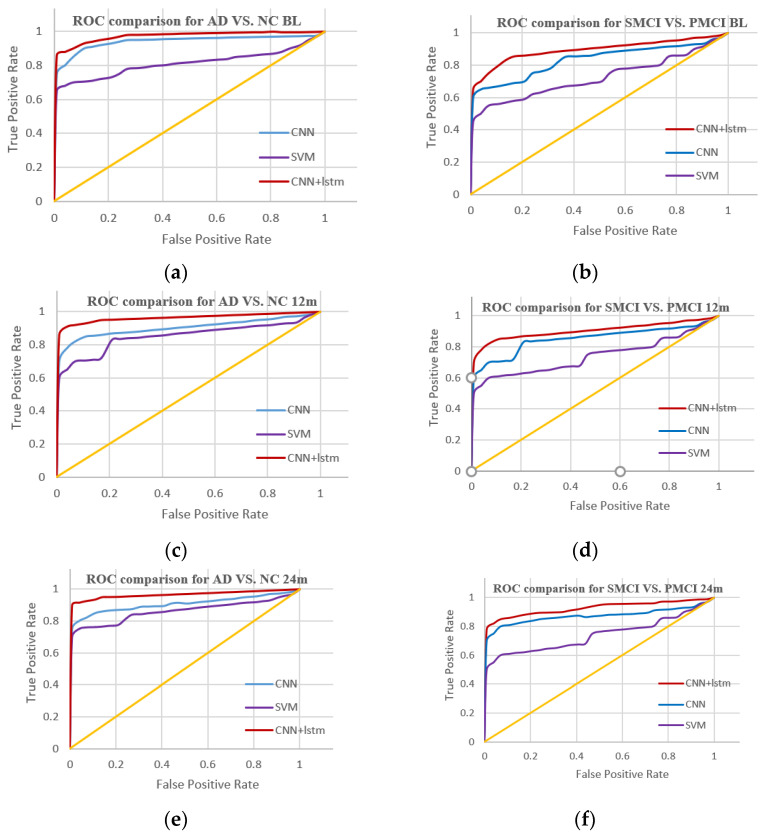
Comparison of the ROC curves of different algorithms; (**a**) comparison of the ROC curves of different algorithms for NC vs. AD at BL; (**b**) comparison of the ROC curves of different algorithms for sMCI vs. pMCI at BL; (**c**) comparison of the ROC curves of different algorithms for NC vs. AD at 12 m; (**d**) comparison of the ROC curves of different algorithms for sMCI vs. pMCI at 12 m; (**e**) comparison of the ROC curves of different algorithms for NC vs. AD at 24 m; and (**f**) comparison of the ROC curves of different algorithms for sMCI vs. pMCI at 24 m.

In this study, the accuracy, precision, and recall of sMCI and pMCI as well as NC and AD groups at the baseline period (BL) and 12 months (12 m) and 24 months (24 m) after the baseline period were compared. As can be seen from the experimental results, there are significant differences between sMCI and pMCI as well as NC and AD samples over time. A comparison of the ROC curves of different algorithms is shown in Figure 13.

## 4. Conclusions

In this paper, we proposed a multi-time model for the diagnosis and prediction of Alzheimer’s disease based on a convolutional neural network and a resting-state fMRI of the brain functional network. The ADNI dataset was used to screen the original fMRI data, and the whole-brain resting-state fMRI of the brain functional network was built after format conversion and image preprocessing. GAN was used to amplify the data as the initial feature of CNN + LSTM, and the model was verified at multiple time points. Compared with other classical algorithms, the experimental results show that the algorithm is effective. AD vs. NC was superior to pMCI vs. sMCI at multiple time points. The diagnosis effect of Alzheimer’s disease using only the SVM model was the worst, and the classification effect of the CNN experiment using only CNN was better than that of SVM at multiple time points. The model based on CNN combined with LSTM proposed by us was superior to the CNN and SVM methods alone in temporal and spatial analyses. This indicates that the spatial and temporal analysis algorithm proposed by us is suitable for the diagnosis and prediction of Alzheimer’s disease.

## Figures and Tables

**Table 1:** Sample demographic information.

Classified	Samples	Sex (Male/Female)	Mean Age	MMSE	CDR
NC-bl	100	50/50	70.80	29.56	0.02
NC-12 m				29.30	0.05
NC-24 m				29.62	0.08
sMCI-bl	75	37/38	72.56	27.56	0.50
sMCI-12 m				26.96	0.52
sMCI-24 m				25.55	0.62
pMCI-bl	72	32/40	75.60	26.52	0.85
pMCI-12 m				24.26	1.22
pMCI-24 m				22.98	2.36
AD-bl	65	30/35	78.20	23.35	3.26
AD-12 m				21.26	3.51
AD-24 m				18.75	3.95

**Table 2:** Based on the experimental results of longitudinal features.

BL	Methods	Time	Accuracy (%)	Precision (%)	Recall (%)
NC VS. AD	SVM	BL	86.1	83.9	80.0
		12 m	87.3	85.5	81.5
		24 m	88.5	84.4	83.1
	CNN	BL	88.5	85.9	84.6
		12 m	89.7	87.5	86.2
		24 m	91.0	89.1	87.7
	CNN + LSTM	BL	90.3	88.9	86.2
		12 m	91.0	87.9	89.2
		24 m	93.3	91.0	92.3
sMCI VS. pMCI	SVM	BL	68.0	66.7	69.4
		12 m	69.4	68.0	70.8
		24 m	70.1	68.4	72.2
	CNN	BL	71.4	69.7	73.6
		12 m	72.1	70.1	75.0
		24 m	73.5	71.4	76.4
	CNN + LSTM	BL	74.1	72.4	76.4
		12 m	74.8	72.7	77.8
		24 m	75.5	73.1	79.2

## Data Availability

Data used in the preparation of this article were obtained from the Alzheimer’s Disease Neuroimaging Initiative (ADNI) database (adni.loni.usc.edu (accessed on 12 January 2022)).
